# Review: microbial transformations of human bile acids

**DOI:** 10.1186/s40168-021-01101-1

**Published:** 2021-06-14

**Authors:** Douglas V. Guzior, Robert A. Quinn

**Affiliations:** 1 Department of Microbiology and Molecular Genetics, Michigan State University, East Lansing, MI 48824 USA; 2 Department of Biochemistry and Molecular Biology, Michigan State University, East Lansing, MI 48824 USA

**Keywords:** Bile acid, Cholic acid, Conjugation, Microbiome, Metabolism, Microbiology, Gut health, *Clostridium scindens*, *Enterocloster bolteae*

## Abstract

Bile acids play key roles in gut metabolism, cell signaling, and microbiome composition. While the liver is responsible for the production of primary bile acids, microbes in the gut modify these compounds into myriad forms that greatly increase their diversity and biological function. Since the early 1960s, microbes have been known to transform human bile acids in four distinct ways: deconjugation of the amino acids glycine or taurine, and dehydroxylation, dehydrogenation, and epimerization of the cholesterol core. Alterations in the chemistry of these secondary bile acids have been linked to several diseases, such as cirrhosis, inflammatory bowel disease, and cancer. In addition to the previously known transformations, a recent study has shown that members of our gut microbiota are also able to conjugate amino acids to bile acids, representing a new set of “microbially conjugated bile acids.” This new finding greatly influences the diversity of bile acids in the mammalian gut, but the effects on host physiology and microbial dynamics are mostly unknown. This review focuses on recent discoveries investigating microbial mechanisms of human bile acids and explores the chemical diversity that may exist in bile acid structures in light of the new discovery of microbial conjugations.

Video Abstract

**Electronic supplementary material:**

The online version contains supplementary material available at 10.1186/s40168-021-01101-1.

## Introduction

### The history of bile

Bile has been implicated in human health for millennia. Hippocrates developed the idea of humourism in the third century BC, which describes the body as being composed of four “humors,” two of which involve bile. When these humors are balanced the body is healthy, but illness occurs when any become unbalanced [1]. Even today, we are still trying to understand how the delicate balance between different bile acid (BA) concentrations throughout the body is associated with states of health or disease. Our gut microbiome, the consortium of microorganisms living in our gastrointestinal system, is a major mediator of BA chemistry and, consequently, the development of healthy or diseased states. For example, abnormally high levels of the microbially modified secondary BA deoxycholic acid (3α, 12α-dihydroxy-5β-cholan-24-oic acid, DCA) is associated with gut dysbiosis and disease [2, 3]. There has been increased research in recent years on the connection between our gut microbiome, BA pool composition, and human health, all of which build on our knowledge from the previous two millennia of BA chemistry. This review will describe discoveries from traditional microbial BA modification pathways and provide context to how the newly discovered microbially conjugated BAs affect our understanding of human bile and its transformation by our microbiome.

### Bile acid biochemistry and physiology

**Fig. 1:**
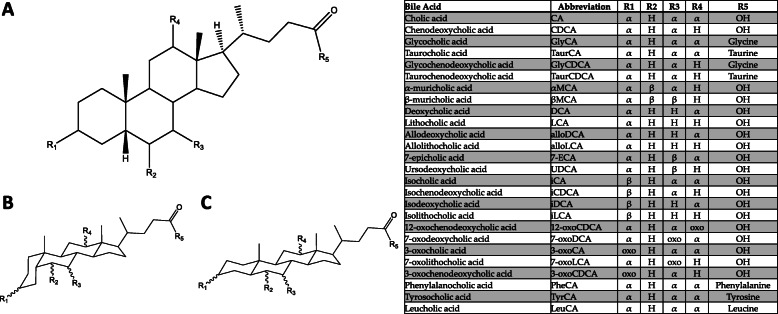
Diversity of known human bile acids. **A** All BAs are built off the same sterol backbone with variations in hydroxylated positions, hydroxyl orientation, and the presence of ketones. CA and CDCA, along with GlyCA, GlyCDCA, TaurCA, and TaurCDCA, make up the primary BA pool. Remaining BAs in the list make up secondary and tertiary BA pools as a result of modifications from gut microbes [5–7]. Allobile acids, although matching in hydroxyl positions to their standard bile acid counterparts, differ in ring orientation. Standard bile acids have the first ring in the **B** transorientation, yielding 5β-BAs, while allobile acids have this ring in the **C** cis-orientation, yielding 5α-BAs

Primary BAs are those synthesized in the liver from cholesterol [4]. The primary BA pool in humans consists of cholic acid (3α, 7α, 12α-trihydroxy-5β-cholan-24-oic acid, CA), chenodeoxycholic acid (3α, 7α-dihydroxy-5β-cholan-24-oic acid, CDCA), and subsequent C24 taurine- or glycine-bound derivatives (Fig. 1). Glycine and taurine bound BAs are also referred to as bile salts due to decreased pK_a_ and complete ionization resulting in these compounds being present as anions in vivo [8–10]. For the purposes of this review, all compounds will be referenced in their protonated form, being named conjugated bile acids in lieu of conjugated bile salts. Primary BAs are heavily modified in the lower gastrointestinal tract to produce a broad range of secondary BAs (Fig. 1). This microbial metabolism is so extensive that instead of primary BAs having the highest prevalence in stool, DCA (a CA derivative) and lithocholic acid (3α-hydroxy-5β-cholan-24-oic acid, LCA, a CDCA derivative), both microbially modified BAs, are the most prevalent [11]. Relevant BAs within humans are not limited to hydroxylation at C3, C7, and C12, but are also found to be hydroxylated at C6 as is the case for α-muricholic acid (3α, 6β, 7α-trihydroxy-5β-cholan-24-oic acid, αMCA) and β-muricholic acid (3α, 6β, 7β-trihydroxy-5β-cholan-24-oic acid, αMCA). Muricholic acids are predominant in mice and scarce in humans, though not absent. MCA forms of bile acids are present in infant urine and feces, though they decrease in concentration to below detectable level in adults [12, 13]. Due to their predominance in mice and rats, MCAs are important in gastrointestinal research using animal models [14].

BAs have traditionally been thought to undergo amino acid conjugation solely in the liver. There is a single human enzyme, bile acid-CoA:amino acid N-acyltransferase (hBAAT), that is responsible for acyl-conjugation. These conjugated primary BAs are secreted via the bile canaliculi into the gallbladder where they are stored until consumption of a meal. They are then secreted into the duodenum and travel through the small intestine, only to be subsequently reabsorbed in the terminal ileum and transported to the liver for re-conjugation if necessary, followed by secretion into the gallbladder and recirculation [15]. This enterohepatic circulation is very efficient, recirculating approximately 95% of secreted bile acids, including some of those modified by the microbiota [16]. The remaining 5% undergoes a myriad of transformations throughout the gastrointestinal tract [5, 17]. Although the specific chemistry of BA reabsorption is not completely elucidated, it is generally understood that conjugated BAs are actively transported by ileal transporters and some passive diffusion across the gut epithelium can occur for both conjugated and non-conjugated BAs, specifically those conjugated to glycine [16, 18]. GlyCA and other glycine conjugates may be able to undergo passive diffusion due to the relatively small change in BA biochemistry caused by glycine conjugation.

BAs play an important role in regulating various physiological systems, such as fat digestion, cholesterol metabolism, vitamin absorption, liver function, and enterohepatic circulation through their combined signaling, detergent, and antimicrobial mechanisms [19]. BAs are agonists of the farnesoid X receptor (FXR), with varying degrees of activity depending on the structure of the compound [20]. CDCA is the most potent FXR agonist, followed by DCA, LCA, and lastly, CA. Though their effects on FXR are less clear and more research is needed, conjugated BAs have also been observed to play a role as FXR agonists, notably within the small intestine where concentrations can reach as high as 10 mM [21, 22]. FXR is responsible for regulating several steps in the synthesis of primary BAs CA and CDCA. The loss of FXR activity in mice results in metabolic perturbations and loss of host BA regulation [23]. FXR plays a major role in protecting the small intestine from overgrowth from the large intestine, regulating key antimicrobial pathways including inducible nitric oxide synthase, IL18, angiogenin production, and production of several antimicrobial peptides, such as those within the *Defa* gene family [21, 24]. TauroBAs, specifically TauroβMCA, have also been shown to act as FXR antagonists, inhibiting BA synthesis via negative regulation [25]. Additionally, BAs are agonists of g-protein coupled receptors such as TGR5 (Takeda G protein-coupled receptor 5) and S1PR2 (sphingosine-1-phosphate receptor 2). S1PR2 is expressed ubiquitously within the liver while TGR5 is expressed primarily in non-parenchymal cells [26]. Expression of both S1PR2 and TGR5 is a balancing act within the liver between homeostasis and damage. S1PR2 is activated by conjugated BAs and results in pro-inflammatory effects that can increase liver damage while TGR5 is activated by all BAs along with several other steroids and results in anti-inflammatory effects in addition to anti-cholestatic and anti-fibrotic effects [26]. These characteristics make S1PR2 inhibitors and TGR5 agonists attractive candidates for drug development.

### Microbial bile acid interactions

Bile acids are potent antimicrobials. As such, they play an important role in the innate immune defense within the intestine. Consequently, modifications of BAs are an essential microbial defense mechanism [27]. BAs have been known to impact susceptible bacteria in both a bacteriostatic and bactericidal fashion since the late 1940s, impacting such genera as *Staphylococcus*, *Balantidium*, *Pneumococcus*, and *Enterococcus* in addition to members of the phylum Spirochaetes [28]. BAs act as detergents in the gut and support the absorption of fats through the intestinal membrane. These same properties allow for the disruption of bacterial membranes. Primary BAs disrupt membranes in a dose-dependent fashion and non-conjugated BAs exact a greater reduction in viability than their conjugated counterparts when tested against *Staphylococcus aureus*, several *Lactobacillus* species, and several *Bifidobacterium* species [27, 29]. As a result of the conjugation to glycine or taurine, primary BAs are fully ionized at physiological pH. While this is important in the movement of BAs from the liver, complete ionization prevents significant interaction and passive diffusion across bacterial membranes whereas non-conjugated CA and CDCA are able to disrupt membranes, cross them, and cause intracellular damage [30]. Conjugated BAs can have more indirect action on the gut microbiota, however, because at high concentrations in the small intestine they modulate FXR and other ileal receptors which control bile synthesis.

### Microbial bile acid transformation pathways

Traditionally, there have been four distinct pathways related to microbial transformations of BAs: deconjugation, dehydroxylation, oxidation, and epimerization. The latter two methods of BA transformations work hand in hand, as formation of oxo-BAs is a key step prior to epimerization. Research into microbial bile salt hydrolases (BSHs) has been the latest boom in health-related BA research since their discovery in the 1970s with over 260 publications listed on PubMed from within the last 10 years (search term ‘bile salt hydrolase’). Additionally, several reviews have been written specifically about the biochemistry, diversity, and implications of microbially transformed BAs on host health [17, 31, 32]. The diversity of BAs has recently been shown to be higher than originally thought as members of the gut microbiota demonstrated the ability to conjugate amino acids to cholic acid independent of the host liver [5].

## Deconjugation

Deconjugation of BAs is considered the “gateway reaction” to further modification [33]. There are several hypotheses that could explain the importance of deconjugation. As previously discussed, deconjugated primary BAs can act as signaling molecules which modify the total bile acid pool, and therefore, the microbiota may have evolved the deconjugation mechanism to manipulate bile production further. Deconjugation also results in increased concentrations of antimicrobial BAs, CA and CDCA, that may drive shifts in microbiome composition and act as a possible form of microbial chemical warfare. BSHs (classified as EC 3.5.1.24) are able to deconjugate both glycine- and taurine-bound primary BAs, though differences in activity may indicate BSH substrate specificity [17]. Members of the gut microbiota may also use the liberated glycine and taurine residues as nutrient sources. Regardless, deconjugation is an essential function of the gut microbiome.

Enzymes capable of catalyzing the deconjugation reaction are found across all major bacterial phyla and within major archaeal species, suggesting that the genes encoding them are horizontally transferable [34, 35]. *Bacteroides* spp. are among one of the phyla suggested to play a major role in deconjugating primary BAs [36]. The diversity of bacteria capable of amino acid hydrolysis includes Gram-positive genera such as *Bifidobacterium* [37], *Lactobacillus* [38, 39], *Clostridium* [40], *Enterococcus* [41], and *Listeria* [42]. However, BSH activity is not limited to Gram-positive bacteria. Gram-negatives such as *Stenotrophomonas* [43], *Bacteroides* [44], and *Brucella* [45] also contribute to amino acid hydrolysis within the gut. In the cases of *Brucella abortus* and *Listeria monocytogenes*, BSH genes are important for virulence and establishing infection within mouse models. A metagenomic study by Jones et al. found BSH-encoding genes are conserved among all major bacterial and archaeal species within the gut [33]. Bacteria capable of BSH activity comprise 26.03% of identified strains of gut bacteria present in humans, although some of these strains may be in low abundance as only 26.40% of BSH-capable strains are present in human guts throughout the globe [46]. The mere ubiquity of BSHs in the gut exemplifies their importance to our microbiota.

**Fig. 2:**
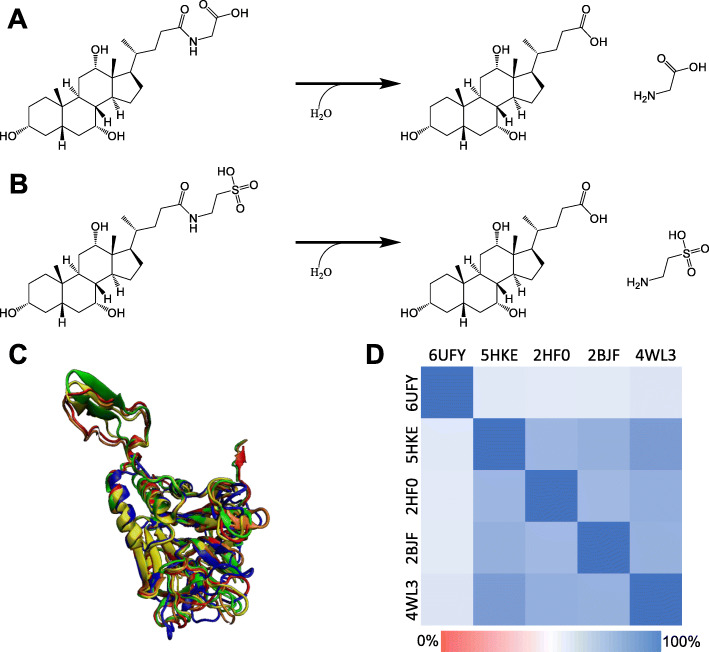
Deconjugation reactions and enzyme homology present between gut bacteria. Regardless of hydroxylation positions, substitution of water for either **A** glycine or **B** taurine yields the same products. **C** Structural homology between subunits from *B. thetaiotaomicron* (6UFY, blue), *L. salivarius* (5HKE, red), *B. longum* (2HF0, yellow), *C. perfringens* (2BJF, green), and *E. faecalis* (4WL3, orange) using Visual Molecular Dynamics (VMD) software [47]. **D** Structural homology (Q_H_) was measured utilizing VMD with a minimum of 0.5804 and a maximum of 0.8533. *E. faecalis* and *L. salivarius* BSHs had the greatest similarity while *B. thetaiotaomicron* was the most dissimilar to all other organisms. These analyses were created de novo for this review

All BSH reactions rely on amide bond hydrolysis in order to free taurine or glycine (Fig. 2A, B). Optimal BSH activity occurs at neutral or slightly acidic pH (5–7) with reported optima around pH 6 [40, 48, 49]. Interestingly, among *Bifidobacterium* spp. arose three separate classes of BSH [37]. Among the three classes of BSH found within *Bifidobacterium* spp., two classes had high activity and differed in substrate specificity. Both classes exhibited a preference for glycine-conjugated BAs but varied in activity for taurine-conjugated BAs. Although BSHs may utilize both taurine and glycine conjugates, encoding many BSHs may allow for slight changes in substrate specificity and more specific manipulation of the bile acid pool. BSH enzymes from *Ligilactobacillus salivarius* (PDB ID: 5HKE) [50, 51], *Bifidobacterium longum* (PDB ID: 2HF0) [52, 53], *Bacteroides thetaiotaomicron* (PDB ID: 6UFY) [54, 55], *Clostridium perfringens* (PDB ID: 2BJF) [56, 57], and *Enterococcus faecalis* (PDB ID: 4WL3) [58] have been crystalized (Fig. 2C). Comparing structural homology (Fig. 2D), *E. faecalis*, *L. salivarius*, *B. longum*, and *B. thetaiotaomicron* each maintained the αββα motif indicating that it is essential for activity [46]. The BSH from *B. thetaiotaomicron* (Fig. 2C, blue) is missing a turn which may be one of the driving factors for the decreased structural homology between the other crystalized BSHs. Analysis of key residues from *L. salivarius*, *B. longum*, *E. faecalis*, and *C. perfringens* amino acid sequences demonstrated highly conserved residues throughout the BSH active site across each genus [46].

## Dehydroxylation at C7

**Fig. 3:**
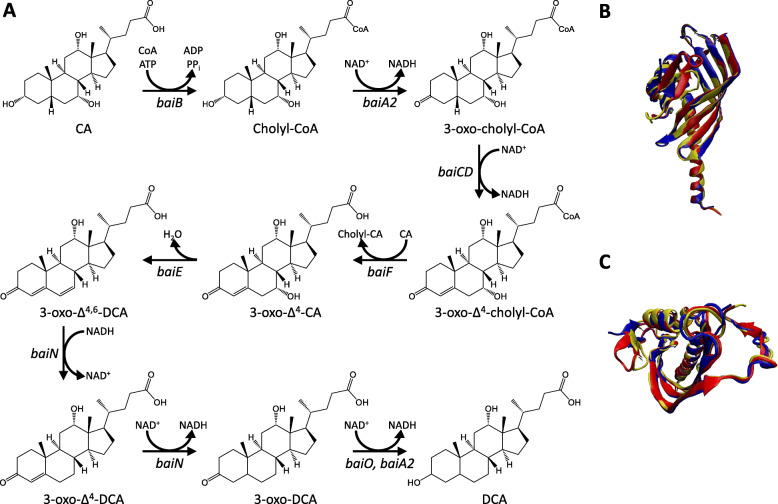
Dehydroxylation pathway for primary BAs CA (R: -OH) and CDCA (R: -H). **A** The pathway to complete 7α-dehydroxylation is a multi-stage process that involves progressive substrate oxidation, likely for molecule stability, prior to dehydroxylation, followed by reduction at each previously oxidized position along the sterol backbone [59]. The enzyme capable of dehydroxylation, BaiE, is highly conserved structurally between *C. scindens* (red), *C. hylemonae* (blue), and *P. hiranonis* (yellow), evident in both **B** side and **C** top-down views of BaiE

One of the key transformations by gut microbes is BA dehydroxylation at C7. Within *Clostridium scindens*, the *bai* operon encodes several proteins needed for the sequential oxidation of CA [59]. The *baiG* gene encodes for a bile acid transporter, allowing for CA uptake. BaiG is also capable of transporting CDCA and DCA [60]. This is followed by CoA ligation in an ATP-dependent manner by BaiB to form cholyl-CoA. Cholyl-CoA is then oxidized twice, first by BaiA and followed by BaiCD, to yield 3-oxo-Δ^4^-cholyl-CoA. BaiF is then hypothesized to transfer CoA from 3-oxo-Δ^4^-cholyl-CoA to CA, yielding 3-oxo-Δ^4^-CA and cholyl-CoA [6]. BaiF CoA transferase activity has already been observed with DCA-CoA, LCA-CoA, and alloDCA-CoA acting as donors and CA acting as an acceptor [59]. The rate limiting step occurs during the dehydroxylation of C7 via BaiE, a 7α-dehydratase. The genes involved in CA 7α-dehydroxylation are capable of recognizing intermediates in the CDCA dehydroxylation pathway as well. Interestingly, CoA-conjugation at C24 was not necessary for dehydratase activity to occur with CA as the substrate, and in some cases enabled for greater k_cat_ and lower K_M_ [61]. Crystal structures of BaiE have been generated in the ligand-absent conformation from *C. scindens* (PDB ID: 4LEH) [62], *Clostridium hylemonae* (PDB ID: 4L8O) [63]*,* and *Peptacetobacter hiranonis* (formerly *Clostridium hiranonis,* PDB ID: 4L8P) [64] (Fig. 3). Each unit displayed structural similarity (Q_H_) greater than 85% as calculated in Visual Molecular Dynamics (VMD) [47]. The enzymes responsible for the reductive arm of BA 7α-dehydroxylation within *C. scindens* are encoded by *baiN*, which is responsible for the sequential reduction of C6-C7 and C4-C5 after dehydroxylation, and by *baiA2*, which catalyzes the NADH-dependent 3-oxoreduction of both 3-oxodeoxycholic acid and 3-oxolithocholic acid [65, 66]. BaiO is proposed to carry out a similar function to BaiA2 in the reductive arm of 7α-dehydroxylation though this has not yet been verified experimentally [6]. 7β-dehydroxylation occurs in a similar fashion, the key difference being that BaiH is used in the place of BaiCD for C4 oxidation [67, 68]. 7β-dehydratase activity is likely the rate limiting step in 7β-dehydroxylation similar to BaiE above, though the exact gene has not yet been identified. This indicates that further research is needed to elucidate the impact and prevalence of organisms capable of 7β-dehydroxylation, especially given the relative absence of 7β BAs.

## Oxidation and epimerization

**Fig. 4:**
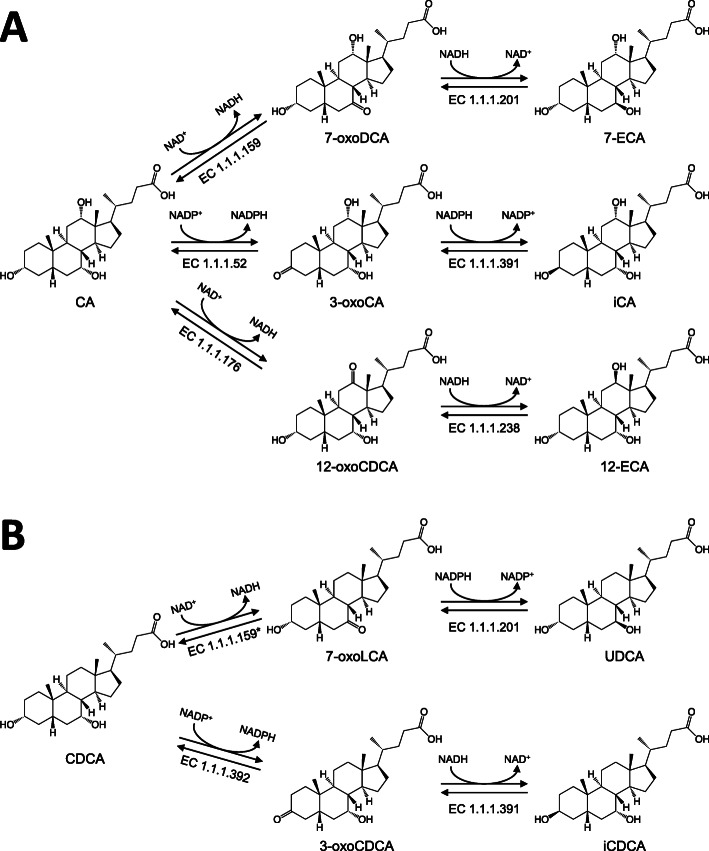
Pathways of CA and CDCA epimerization, including corresponding EC identifiers. **A** CA undergoes three different epimerization pathways leading to the production of iCA (via 3α/β-HSDH), UCA (via 7α/β-HSDH), or 12-ECA (via 12α/β-HSDH) while **B** CDCA undergoes two distinct epimerization pathways leading to the production of UDCA (via 7α/β-HSDH) or iCDCA (via 3α/β-HSDH). *S. maltophilia transforms CDCA to 7-oxo-CDCA but the enzyme is categorized under EC 1.1.1.159, where the official reaction involves CA 7α-oxidation [70]

Epimerization of BAs is carried out by gut microbes and further diversifies the chemistry of secondary BAs. This occurs in two distinct steps: oxidation of the hydroxyl group by a position-specific hydroxysteroid dehydrogenase, such as a 7α-HSDH, followed by the reduction of another position-specific hydroxysteroid dehydrogenase, 7β-HSDH. Both reactions do not need to be carried out by the same organism and co-cultures of microbes are known to possess epimerization capabilities [69]. CA can be epimerized to form derivatives such as ursocholic acid (3α, 7β, 12α-trihydroxy-5β-cholan-24-oic acid, UCA), 12-epicholic acid (3α, 7α, 12β-trihydroxy-5β-cholan-24-oic acid, 12-ECA), or isocholic acid (3β, 7α,12α-trihydroxy-5β-cholan-24-oic acid, iCA) (Fig. 4A), while CDCA can be epimerized to form either UDCA or isochenodeoxycholic acid (3β,7α-Dihydroxy-5β-cholan-24-oic acid, iCDCA) (Fig. 4B). Both oxidation and subsequent epimerization have been observed at all three CA hydroxyl positions as well as both CDCA hydroxyl positions and are responsible for much of the diversity found in non-conjugated BAs.

Recently, *C. scindens*, *C. hylemonae*, *C. perfringens*, and *P. hiranonis* have all been observed to produce enzymes capable of hydroxysteroid 3α-dehydrogenation, an important step in the pathway toward 7α-dehydroxylation [35]. However, unlike *C. scindens*, *C. hylemonae*, and *P. hiranonis*, *C. perfringens* has not been reported to produce LCA or DCA and its growth is inhibited by both secondary BAs [71]. 3α-dehydrogenation also occurs outside of the genus *Clostridia* and includes other intestinal organisms such as *Blautia producta* and *Eggerthella lenta* (formerly *Eubacterium lentum*) in addition to environmental species such as *Acinetobacter lwoffii* [65, 72, 73]. Surprisingly, *E. lenta* 3α-HSDH is capable of utilizing both TaurBAs and GlyBAs as substrates and in the case of CDCA oxidation, 3α-HSDH activity increased when conjugated forms of CDCA were used as substrates [74]. This goes against the notion that bile BA deconjugation is the essential ‘gateway’ reaction and further investigation is required to elucidate if glycine and taurine residues impact molecular mechanisms of catalysis in addition to if conjugated BA oxidation impacts subsequent transformations. 7α-epimerization to UDCA occurs in the gut by members such as *Clostridium baratii* among other isolates not yet identified [75, 76]. *C. baratii* has been shown to epimerize CDCA to UDCA but was not capable of epimerizing glyco- and tauro-BAs and instead deconjugated TaurCDCA prior to epimerization [75]. Epimerization of CDCA, independent of conjugation, is important for producing the protective BA UDCA. *Ruminococcus gnavus*, *Clostridium absonum*, *Stenotrophomonas maltophilia*, and *Collinsella aerofaciens* all contribute to the UDCA pool via conversion of 7-oxo-LCA in an NADH or NADPH-dependent fashion [70, 77–79]. Optimum pH varied between species; *C. absonum* 7β-HSDH functioned optimally at pH 8.5 while *R. gnavus* and *C. aerofaciens* functioned optimally at pH 6. 12β-HSDH activity can occur in both acidic and alkaline conditions. *R. gnavus*, in contrast to *C. absonum* and *C. aerofaciens*, displayed a clear preference in catalyzing the conversion of 7-oxo-LCA to UDCA with a specificity constant 55-fold higher than that of the conversion of UDCA to 7-oxo-LCA [77]. This directionality of activity paired with the protective properties of UDCA support *R. gnavus* as a potential probiotic, and this role should be further investigated.

Several gut bacteria have recently been identified to produce 12α-hydroxysteroid dehydrogenases (12α-HSDH). *E. lenta* demonstrates 12α-HSDH capabilities in addition to 3α-HSDH. *E. lenta* 12α-HSDH has an estimated molecular weight of 125 kDa and has a broad pH optimum, between pH 8 and 10.5 [80, 81]. Catalysis requires NAD^+^ or NADP^+^ as a cofactor, though there is a preference for NAD^+^ [65, 81]. *E. lenta* 12α-HSDH reaction velocity increased when tested with methylated BAs, suggesting a preference for hydrophobic BAs [80]. Similar to its 3α-HSDH, *E. lenta* 12α-HSDH is capable of utilizing both glycine- and taurine-bound BAs [74]. *Enterorhabdus mucosicola* is also capable of both 3α and 12α oxidation, although 12α-HSDH activity is limited to when C7 position has already been oxidized [82, 83]. *C. scindens*, *P. hiranonis*, and *C. hylemonae* have since been reported to produce 12α-HSDHs and it is hypothesized that *Bacteroides* species also encode 12α-HSDHs [35, 65]. Across all three clostridial species, there was a robust preference for 12-oxo-LCA over 12-oxo-CDCA suggesting the C7 hydroxyl group, or lack thereof, plays a large role in determining enzyme activity. Oxidation at C12 occurs for 12β BAs as well and has been observed in strains of *Clostridium paraputrificum*, *Clostridium tertium*, and *Clostridioides difficile* [84]. These 12β-HSDHs are relatively stable at physiological conditions, maintaining activity at 37 °C for approximately 45 minutes at pH 8.5 [85]. Based on the findings by Edenharder and Pfutzner, *C. paraputrificum* 12β-HSDH behaves in a similar manner to established 12α-HSDHs, as shown by its pH optimum and molecular weight. The gene encoding the 12β-HSDH in *C. paraputrificum* was recently identified, allowing for investigation into the diversity of potential 12β-HSDH producers [86]. Putative 12β-HSDH genes were found across Firmicutes, Actinobacteria, and Alphaproteobacteria. However, there may be several forms of 12β-HSDH as the authors did not find homologs to the *C. paraputrificum* 12β-HSDH in *C. difficile* and *C. tertium* even though both species are capable of 12β-HSDH activity.

Members of the gut microbiota are not only capable of reducing BAs with a single position oxidized, but some also reduce BAs oxidized at two or three positions. Similar trends regarding non-target hydroxyl oxidation have been observed by other *Coriobacteriaceae,* such as *C. aerofaciens*, *E. lenta*, and *Lancefieldella parvula* (formerly *Atopobium parvulum*) [82]. Not all members oxidized DCA at both C3 and C12 independent of the other position, but all of the strains observed to modify DCA were shown to oxidize at both positions [82]. *C. scindens* and *P. hiranonis* were among the only bacteria capable of completely hydrogenating 3,7,12-trioxolithocholic acid, a fully oxidized derivative of CA, to CA [35]. Oxidation may be a way for microbes to detoxify BAs. By decreasing their amphipathicity, oxidized BAs progressively lose the ability to act as detergents, preventing DNA and membrane damage.

## Reconjugation: microbially conjugated bile acids

A novel set of recently discovered BAs were conjugated at the C24 acyl site similarly to the host conjugation mechanism [5]. Instead of the traditional amino acids taurine and glycine, these compounds were conjugated with the amino acids phenylalanine, leucine, and tyrosine on a cholic acid backbone. The initial work associated these molecules with the gut microbiota and follow-up experiments identified the bacterium *Enterocloster bolteae*, formerly *Clostridium bolteae***,** as a species responsible for their production. In light of their microbial origin and the mechanism mirroring that of host-conjugation, we hereby refer to these compounds as “microbially conjugated bile acids” (MCBAs).

The exact mechanism of this microbially mediated conjugation has yet to be elucidated, although it may rely on a similar mechanism to hBAAT within the liver involving a Cys-Asp-His triad, with cysteine functioning as the catalytic residue for nucleophilic attack [87]. Regardless of their mechanism of production, the addition of unique amino acid chemistry on the BA acyl-site inevitably modifies its chemical properties. Phenylalanine, a large hydrophobic amino acid, will greatly increase the hydrophobicity of the BA itself and possibly induce steric hindrance to any binding mechanisms with ileal receptors or BA transporters. Leucine, too, is a relatively large hydrophobic residue, which may create similar chemical properties to that of phenylalanine. The additional hydroxyl group on the aromatic ring of tyrosine may create some unique properties as this will increase the compound’s hydrophilicity and create a more polar hydrophilic BA, similar to the increase in polarity provided by taurine conjugation to cholic acid. The presence of any of these amino acids at the conjugation site will also alter the BA’s emulsifying properties, as a primary function of these compounds is to solubilize fat from our diet. Although not yet shown in the literature, it is likely that the diversity of MCBAs will increase due to the plethora of amino acid residues available for conjugation and the immense microbial diversity present in the human gut. Until the mechanism of their synthesis is enzymatically elucidated and exhaustive searches into MCBA diversity are performed, our knowledge of the limits on amino acid conjugation of BAs by the human microbiota remains incomplete.

The functions of phenylalanine, leucine, and tyrosine CA conjugates remain mostly unknown, though gavage of mice with these compounds has been shown to result in agonism of FXR. Further investigation into the roles of known and unknown BA conjugates may yield novel drug targets or therapeutic agents for the treatment of numerous enteric diseases. Evidence already points toward BA hydrophilicity playing a major role in activity of several BA modifying enzymes; the three novel conjugates currently reported represent three of the four most lipophilic amino acids based on partition coefficient [88]. Thus, identifying organisms responsible for conjugation of other amino acids to other BAs and amino acid-specific mechanisms are the necessary first steps to determining how microbes are utilizing these compounds to impact the host or competing members of the microbiota.

## Molecular diversity of microbially conjugated bile acids

**Fig. 5:**
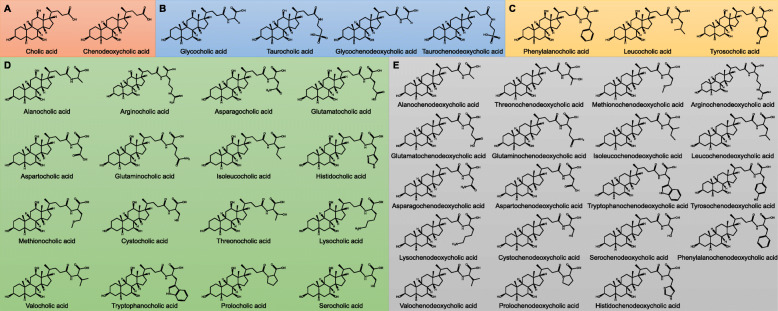
Potential increased diversity of host BA pool as a result of MCBA production. With the current understanding of BA metabolism, **A** primary BAs CA and CDCA are known to be conjugated in the liver to taurine and glycine to form **B** GlyCA, TaurCA, GlyCDCA, and TaurCDCA, completing the pool of primary human BAs. In light of recent research, CA is also known to be conjugated by gut microbes to form **C** PheCA, LeuCA, and TyrCA [5]. Expanding the potential library of microbially conjugated BAs by including the remaining amino acids conjugates for **D** CA and **E** CDCA increases the diversity of human BAs over 5-fold for these backbones alone

Over 140 amino acids are known to occur in natural proteins [89]. The human BA pool consists of a sterol backbone capable of hydroxylation at four different positions (including C_6_, observed in MCA), which can be ɑ- or β-hydroxylated, oxidized to form a ketone, or absent. This backbone can also be present as one of two stereoisomers: 5ɑ-sterol or 5β-sterol, significantly broadening potential BA diversity. Limiting the bile acid backbone to only those known to be conjugated by the host (CA and CDCA) in addition to limiting the amino acid conjugated to those naturally occurring in humans, the potential diversity of the human conjugated BA pool increases over 5-fold from what is currently known (Fig. 5). This estimate does not consider non-amino acid conjugates, such as ciliatocholic acid or cholyl-CoA, nor does it include the diversity of potential host hydroxyl modifications, such as sulfonation [90, 91]. Overall, the human bile acid pool is dominated by CA, CDCA, and DCA [92]. Subsequent taurine and glycine conjugation increases this pool to 9 BAs. Limiting the estimate of possible BA-amino acid conjugates to standard amino acids and the three BAs listed above increases the potential human BA pool to 66 unique conjugates. Finally, including all potential oxidized, epimerized, and dehydroxylated states of each hydroxyl group present on CA (C3, C7, C12) in addition to ring orientation expands the number of potential human BA conjugates to over 2800. Although it is unlikely that the number of physiologically relevant MCBAs is this high, one can imagine the potential diversity of MCBAs and the potential for their impact on the gut microbiota and the host.

Thus far, only relatively hydrophobic amino acids have been reported to be conjugated to cholic acid by microbes, lowering the overall partition coefficient of each molecule. The partition coefficient is the log-ratio of concentrations of a compound in a hydrophobic solvent, such as 1-octanol, compared to a hydrophilic solvent, such as water. This is to say that a higher value indicates that the compound is more present in the hydrophobic phase rather than the hydrophilic phase. As expected, hydrophobicity increases with the reduction of BAs. CA has a partition coefficient of 2.02, which increases to 3.28 when CA is reduced to CDCA and further increases to 3.5 when reduced to DCA [93]. The conjugation of both glycine and taurine to any sterol significantly increases the hydrophilicity of the compound, thus decreasing the partition coefficient for each BA. Therefore, the acyl-conjugation of BAs undoubtedly affects their function. Similarly, microbial conjugation with hydrophobic amino acids would also affect their detergent, signaling, and antimicrobial properties, as well as BA transport. One may wonder then, why do gut microbiota conjugate our bile acids? There are a multitude of possible explanations ranging from enzyme promiscuity to antimicrobial metabolite production, to targeted manipulation of the host BA signaling and regulatory system. Only further research on the genetic, biochemical, and microbiological characterization of the conjugation mechanism and its microbial and host effects will provide the answers. Nevertheless, the MCBA chemical diversity already detected in the mammalian gut, and the potential described above, will invariably diversify the chemical properties of the bile acid pool.

## Microbial bile acid products and host health

Though BAs themselves function as important antimicrobial agents, microbial modification of BAs is equally important in disease prevention and maintenance of a healthy gut microbiome. Though *C. difficile* infections are devastating, fecal microbiota transplant can be a successful treatment in some cases. Successful transplants correlate with an increase in BSH copy number compared to levels prior to transplant, suggesting that microbial modifications of primary BAs play a role in protecting the host against microbial infection [94]. The host microbiota plays an important role in protection against colonization by pathogenic organisms, and the involvement of BA modification in this protective effect is only beginning to be understood [95]. Decreased bile acid deconjugation correlates with several irritable bowel diseases, such as ulcerative colitis, Crohn’s disease, and irritable bowel syndrome [34]. Supplementing diets with microbially transformed BAs can have profound beneficial effects on host pathology. LCA production, a result of CDCA dehydroxylation, is one of the more interesting transformations by gut microbes with a known impact on host health. LCA has been observed to act as an anti-inflammatory agent and protect against colitis in a mouse model [96]. However, LCA and DCA, another the secondary BA, are known carcinogens. While primary human bile acids are known to induce DNA damage within bacteria, LCA and DCA have been observed to damage DNA within mammalian cells [97]. DCA exposure has also been correlated with increased apoptosis and increased production of reactive oxygen and nitrogen species. Subsequently, LCA and DCA are the most prevalent bile acids in human colorectal cancer [98].

Epimerized BAs also influence host health. UDCA, the 7β epimer of CDCA, exhibits protective effects in the gut, specifically through inhibition of TNFα, IL-1β, and IL-6 release [96]. UDCA use has been shown to counteract the apoptotic effects of DCA [99]. UDCA has also been approved for use in gallstone dissolution and in treating primary biliary cholangitis, the later indication as a result of the ability of UDCA to increase bile acid biosynthesis [96, 99]. One caveat of UDCA use is that, at high doses (28–30 mg/kg/day), long-term use leads to increased risk of colorectal cancer in patients with ulcerative colitis and primary sclerosing cholangitis [100].

It is possible that MCBAs may also play a role in disease mechanisms, as PheCA, TyrCA, and LeuCA were more prevalent in patients with inflammatory bowel disease and cystic fibrosis (and though not disease related, were also found in infants) [5]. However, one cannot know, simply by detection in a diseased population, whether MCBAs, or any BA for that matter, are cause or consequence of a particular diseased state; a conundrum that is well known in the microbiome field. There is evidence that at least one microbe that produces MCBAs, *E. bolteae* (referred to as *C. bolteae* in the referenced manuscript), may be involved in severe IBD and Crohn’s disease, as it was identified as one of the most transcriptionally active microbes in the dysbiotic and diseased gut and MCBAs were elevated in these same samples [5, 101]. This association indicates that MCBAs may be involved in severe IBD, but future research is required. Regardless, BAs can serve as markers for various disease states [97, 98] and can themselves be used as therapeutics, such as in the case of UDCA, making them an important group of compounds for identification and treatment of human disease.

## Conclusions

Although BAs have been studied for centuries, recent discoveries show that we still have much to learn. The host BA pool controls microbial diversity, but so too does microbial metabolism of these BAs drive host physiology. In this sense, BAs act as the language of an intricate molecular cross-talk between humans and their gut microbiota. Mechanisms of microbial modification of host BAs continue to be elucidated as do the roles BA metabolism plays in host health. The presence of MCBAs in the human BA pool demonstrates the need for further study of microbial BA modification and further expands the chemical language our gut microbiota uses to communicate with its host.

## Data Availability

Not applicable

## Abbreviations

BA: Bile acid
CA: Cholic acid
12-ECA: 12-epicholic acid
iCA: Isocholic acid
UCA: Ursocholic acid
HCA: Hyocholic acid
αMCA: α-muricholic acid
βMCA: β-muricholic acid
CDCA: Chenodeoxycholic acid
UDCA: Ursodeoxycholic acid
iCDCA: Isochenodeoxycholic acid
DCA: Deoxycholic acid
HDCA: Hyodeoxycholic acid
LCA: Lithocholic acid
GlyCA: Glycocholic acid
TaurCA: Taurocholic acid
GlyCDCA: Glycochenodeoxycholic acid
TaurCDCA: Taurochenodeoxycholic acid
PheCA: Phenylalanocholic acid
TyrCA: Tyrosocholic acid
LeuCA: Leucholic acid
HSDH: Hydroxysteroid dehydrogenase
BSH: Bile salt hydrolase
hBAAT: Human bile acid-CoA:amino acid N-acyltransferase
FXR: Farnesoid X receptor
